# Shumian Capsule Improves the Sleep Disorder and Mental Symptoms Through Melatonin Receptors in Sleep-Deprived Mice

**DOI:** 10.3389/fphar.2022.925828

**Published:** 2022-07-08

**Authors:** Wenhua Li, Yinlong Cheng, Yi Zhang, Yazhi Qian, Mo Wu, Wei Huang, Nan Yang, Yanyong Liu

**Affiliations:** ^1^ Department of Pharmacology, Institute of Basic Medical Sciences, Chinese Academy of Medical Sciences and School of Basic Medicine, Peking Union Medical College, Beijing, China; ^2^ Medical College, Tibet University, Lhasa, China

**Keywords:** sleep disorder, sleep deprivation, Shumian capsule, traditional Chinese medicine, melatonin receptor

## Abstract

Healthy sleep is vital to maintaining the body's homeostasis. With the development of modern society, sleep disorder has gradually become one of the most epidemic health problems worldwide. Shumian capsule (SMC), a kind of traditional Chinese medicine (TCM) commonly used for insomnia, exhibits antidepressant and sedative effects in clinical practice. However, the underlying mechanisms have not been fully clarified. With the aid of a network pharmacology approach and function enrichment analysis, we identified the involvement of melatonin receptors in the antidepressant and sedative effects of SMC. In sleep-deprived mice, SMC treatment significantly alleviated insomnia and relevant mental alterations by improving both sleep latency and sleep duration. However, ramelteon, a selective melatonin receptor agonist that has been approved for the treatment of insomnia, only improved sleep latency. Additionally, SMC exhibited comparable effects on mental alterations with ramelteon as determined by an open-field test (OFT) and forced swimming test (FST). Mechanistically, we revealed that the melatonin receptor MT1 and MT2 signaling pathways involved the therapeutic effects of SMC. In addition to the single effect of traditional melatonin receptor agonists on treating sleep onset insomnia, SMC had therapeutic potential for various sleep disorders, such as sleep onset insomnia and sleep maintenance insomnia. Convergingly, our findings provide theoretical support for the clinical application of SMC.

## 1 Introduction

As one of the most fundamental physiological activities, sleep is essential for many physiological functions, such as energy conservation, modulation of the immune system, brain waste clearance, cognition, and memory (Zielinski et al., 2016). With increasing social competition, people spend more time on studies and work, which always causes chronic sleep deprivation and subsequent health crises. At present, almost a quarter of the population in the world has been plagued by sleep disorders (Fang et al., 2019). Sleep disorders involve various aspects and patterns of sleep difficulties, which are often complicated by mental disorders, including post-traumatic stress disorder, major depression disorder, generalized anxiety disorder, and bipolar disorder (Smith et al., 2005).

Hypnotics are the main strategy of pharmacological intervention to treat sleep disorders. So far, commonly used hypnotics include benzodiazepines and non-benzodiazepines, as well as other medications such as melatonin receptor agonists, orexin receptor antagonists, antidepressants, and antihistamines (Feng et al., 2018; Pavlova and Latreille, 2019). However, benzodiazepines and non-benzodiazepines, antidepressants, and antihistamines have shown various side effects, such as headache, drowsiness, dizziness, grogginess, and withdrawal symptoms (Sateia et al., 2017; Feng et al., 2018). Melatonin receptor agonists (e.g., ramelteon) and orexin receptor antagonists (e.g., suvorexant) represent a new class of medications for the treatment of insomnia by acting on their respective receptor sites, but they were reported to be only applicable to treat specific types of sleep disorder (Sateia et al., 2017).

Traditional Chinese medicine (TCM) is usually processed with several combined herbs, whose therapeutic efficacy has been well recognized worldwide. In contrast to chemical drugs, multi-drug combinations and multi-target interaction endow TCM with a complex system of medical theory and practice, which is supposed to maximize their therapeutic efficacy by inducing synergic effects and simultaneously reducing possible side effects (Zhang Y et al., 2020). Shumian capsule (SMC) is a kind of TCM capsule, mainly composed of eight herbs: Ziziphi Spinosae Semen, Bupleuri Radix, Paeoniae Radix Alba, Albiziae Flos, Albiziae Cortex, *Bombyx batryticatus*, Cicadae Periostracum, and Junci Medulla. According to the theory of Traditional Chinese Medicine, Ziziphi Spinosae Semen is the sovereign medicinal, which nourishes the heart and emolliates the liver to tranquilize. Bupleuri Radix soothes the liver and removes qi stagnation, while Paeoniae Radix Alba nourishes the blood and emolliates the liver; both are the minister medicines. Albiziae Flos and Albiziae Cortex remove the depression to tranquilize; Cicadae Periostracum dispels wind, arrests convulsions, and removes phlegm to tranquilize; *B. batryticatus* disperses wind and heat to tranquilize; those four ingredients are the assistant medicines. In addition, Junci Medulla clears heart fire serving as the assistant and courier medicinal. All the ingredients in the formula combined possess the actions of soothing the liver, removing depression, and nourishing the heart to tranquilize. Several clinical studies have reported that SMC safely improves sleep quality in insomnia patients with depression and anxiety, and significantly alleviates the symptoms of insomnia, anxiety, and depression in convalescent patients of COVID-19 infection (Li et al., 2021; Wang et al., 2021; Chen et al., 2022). A systematic review and meta-analysis also confirmed the safety of SMC in the clinical application (Wang et al., 2021). However, its underlying molecular mechanisms remain unclear.

Currently, network pharmacology has become a novel and powerful solution for TCM drug evaluation and mechanism research (Zhang et al., 2019). In this study, we performed a protein-protein interaction (PPI) analysis on the intersection targets of SMC and insomnia predicted by the STRING database and constructed the drug component target disease network diagram. The function enrichment analyses were also adopted to explore the neuroregulatory activities of SMC. Our findings demonstrated that SMC improved sleep disorder through melatonin receptors (MT) MT1 and MT2 in sleep-deprived mice.

## 2 Materials and Methods

### 2.1 Network Pharmacology Analysis

#### 2.1.1 Identifying Targets From Active Ingredients of SMC

The active ingredients and targets of SMC were obtained using the traditional Chinese medicine systems pharmacology database and analysis platform (TCMSP) (https://tcmsp-e.com/) (screening conditions: the bioavailability (OB) ≥ 30% and drug-like properties (DL) ≥ 0.18). The data of Ziziphi Spinosae Semen, Bupleuri Radix, Paeoniae Radix Alba, and Junci Medulla were obtained from TCMSP. Partial active ingredients and targets of Albiziae Flos and Albiziae Cortex were got from the ETCM database (https://tcmip.cn/). The active ingredients of Albiziae Flos, Albiziae Cortex, *B. batryticatus*, and Cicadae Periostracum were supplemented by the Chemistry Professional Database of Chinese Academy of Sciences (http://www.organchem.csdb.cn [1978-2016]), traditional Chinese medicine database YATCM (http://cadd.pharmacy.nankai.edu.cn/yatcm/) and related article report, while the potential targets were predicted by SwissTargetPrediction website (http://swisstargetprediction.ch/). The file “drug” was generated by screening out the unqualified active ingredients (screening conditions: the bioavailability (OB) ≥ 30% and drug-like properties (DL) ≥ 0.18) and converting the target information into gene symbols in R software.

#### 2.1.2 Searching Targets of Insomnia

The insomnia-related protein targets were screened through the Uniprot database (https://www.uniprot.org/) using the keyword “insomnia” and setting the species as “human”. The file “disease” was generated by downloading the screened genes.

#### 2.1.3 Predicting the Potential Targets of SMC for Insomnia Therapy

The target gene data of diseases and drugs were imported into the software “R for Windows.” The overlapping targets among compound and insomnia targets were used to draw a Venn diagram and generate the file “drug-disease.”

#### 2.1.4 Protein–Protein Interaction Network Construction

The protein interaction relationship file was obtained by importing the intersection targets into the STRING (https://string-db.org/) database. Then, the PPI network diagram of drugs for the treatment of insomnia was obtained by importing the protein interaction relationship file into Cytoscape V3.8.0. The top 30 genes were plotted with the software “R for Windows” based on the degree.

#### 2.1.5 Gene Ontology and Kyoto Encyclopedia of Genes and Genomes (KEGG) Pathway Enrichment Functional Analysis

We installed the “Bioconductor” packages in R software, set the *p* value <0.05 as the threshold, and ran the program to draw a bubble chart. The top 20 pathways of GO and KEGG enrichment analyses were sorted based on the *p* value.

### 2.2 Experiment Verification

#### 2.2.1 Animals

Male C57BL/6 mice of SPF grade, 8 weeks old (week, W), were purchased from SPF Biotechnology Co., Ltd. (Beijing). The mice were housed in the environment with a normal light-dark cycle (12 h of light and 12 h of dark every day), with a controlled temperature at 24 ± 2°C and 50% constant humidity. This study was conducted in accordance with procedures approved by the Institutional Animal Care and Use Committee at Peking Union Medical College (Beijing, China) (Ethics No. ACU​C-A02-2022-073).

#### 2.2.2 Establishment of a Mouse Model of Sleep Deprivation and Drug Treatment

Mice were maintained in the sleep deprivation box with 24 platforms which were 5 cm high, 3 cm in diameter, and remained 5 cm apart from others. Food and water were placed in the food trough on the lid, allowing the mice to move and eat freely. The water temperature around the platform was about 22°C, with platforms 1 cm above the water surface. Then, the mouse was individually placed on each platform. Drug treatment was performed everyday at 9 a.m. After 4-h rest, the mice were then subjected to chronic sleep deprivation for 20 h. The sleep deprivation procedure lasted for 4 weeks, and then the behavioral experiments were performed.

In the experiment of pharmacotherapeutic verification, mice were divided into six groups with 15 mice each: control, model, Ra, SMC-L, SMC-M, and SMC-H. Except for the control group, other groups were subjected to the sleep deprivation procedure. The Ra group was given 1 mg/kg (i.g.) ramelteon, while the SMC-L, SMC-M, and SMC-H groups were given 0.25 g/kg, 0.5 g/kg, and 1 g/kg (i.g.) powder of SMC respectively.

In the experiment of target verification, mice were divided into six groups of 15 mice each: control, model, Ra, SMC, SMC + LU, and SMC+4-P. Except for the group control, other groups were accepted for the sleep deprivation. The Ra group was given 1 mg/kg ramelteon every day, while the SMC, SMC + LU, and SMC+ 4-P groups were given 1 g/kg (i.g.) powder of SMC every day. Meanwhile, SMC + LU group and SMC+ 4-P group were also simultaneously given 5 mg/kg (i.p.) luzindole or 3 mg/kg (i.p.) 4-P-PDOT respectively every day, where luzindole is non-selective MT and 4-P-PDOT a selective MT2 antagonist (Boutin et al., 2020).

#### 2.2.3 Pentobarbital Sodium Righting Reflex Test

Mice were placed on their backs following an injection of 1% pentobarbital sodium (42 mg/kg, i.p.) selected as the dose to induce complete sleep and then monitored the mice for signs of sleeping. Our standard for sleep was that the mice lost their righting reflex for more than 1 min. Sleep latency was defined as the time between the injection of pentobarbital sodium and the beginning of losing righting reflex loss. Sleep duration was defined as the time from the beginning to the recovery of righting reflex loss (Bian et al., 2021).

#### 2.2.4 Open-Field Test

OFT was employed to assess the mental state of mice as we previously described (Wei et al., 2020; Cao et al., 2021). The open-field apparatus consisted of a square arena (40 × 40 × 38 cm), divided into 16 same small squares with lines. The 4 squares in the center of the testing area were defined as a center field, while the other 12 squares were defined as a periphery field. After adapting to the experimental environment for 2 h, each mouse was placed in the periphery field of the area and monitored for 5 min with an overhead video tracking system (Ethovision XT 10, Noldus Information Technology BV, Wageningen, the Netherlands). The time and distance traveled in the center area were measured. The box was cleaned with ethanol after each mouse.

#### 2.2.5 Forced Swimming Test

FST was performed as previously described (Wei et al., 2020), mice were placed individually into a glass cylinder (30 cm in height and 18 cm in diameter) filled with 15 cm deep water and videotaped for 6 min. The immobility time was defined when the mice floated in a motionless position for the last 4 min without additional activity other than the necessary movements for the mice to keep their heads above the water.

#### 2.2.6 Western Blotting Analysis

Protein was extracted from hypothalamus tissues after behavioral experiments, followed by the determination of a total protein and western blotting as reported in our previous research (Cao et al., 2021). The relative protein levels were determined by the intensity of bands using ImageJ software (National Institutes of Health) and normalized against the internal control β-actin. Antibodies against β-actin (Cat. No. 4970), clock (Cat. No. 5157), CaMKⅡ (Cat. No. 3362), and p-CREB (Ser133) (Cat. No. 9198) were purchased from Cell Signaling Technology (Danvers, MA, United States). Antibodies against MT1 (Cat. No. SC-390328) were purchased from Santa Cruz Biotechnology (Santa Cruz, CA, United States). Antibodies against MT2 (Cat. No. ab203346) were purchased from Abcam (Cambridge, United Kingdom). Antibodies against OX1R (Cat. No. AOR-001) and OX2R (Cat. No. AOR-002) were obtained from Alomone Labs (Jerusalem, Israel).

#### 2.2.7 Statistical Analysis

All the data in our results were presented as mean ± SD. For the multi-group comparisons, the data were analyzed by one-way analysis of variance. *p* < 0.05 was considered statistically significant.

## 3 Results

### 3.1 The Predictive Targets of SMC Components for Insomnia Therapy

**FIGURE 1:**
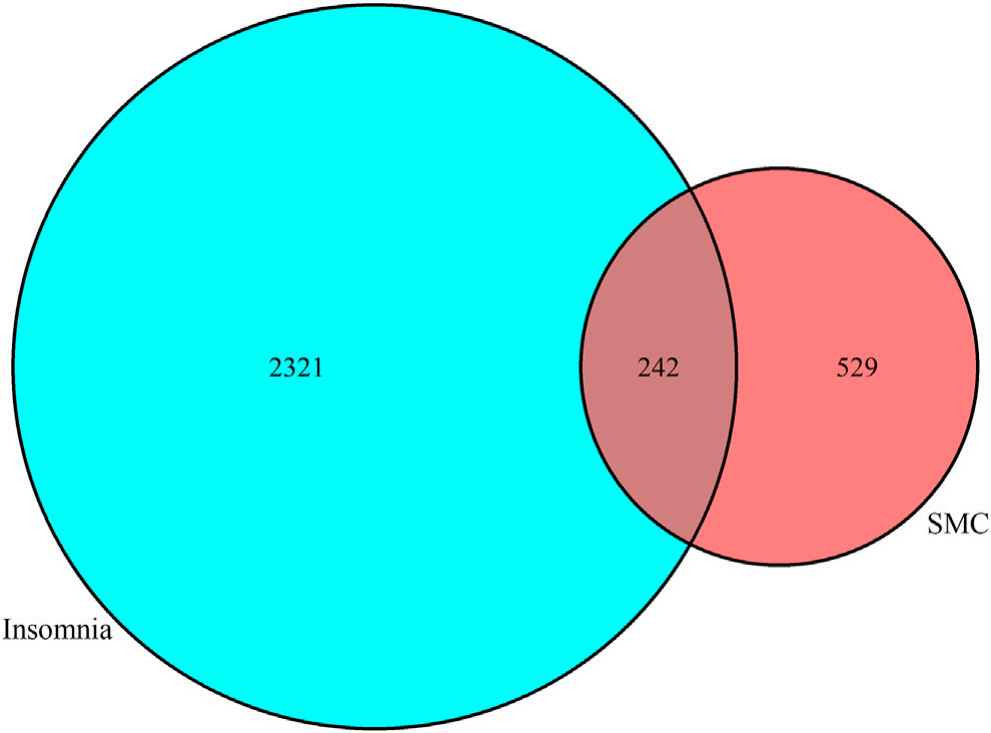
Venn diagram revealing 242 common potential therapeutic targets between SMC and insomnia.

The predictive targets activated by components of SMC were searched from the database of TCMSP and ETCM. For the components without any annotated targets, we drew their chemical formulas using the software ChemDraw, obtaining their predictive targets on the SwissTargetPrediction website. Meanwhile, the insomnia targets were searched from the Uniprot database. The obtained predictive targets were shown in the Venn diagram (Figure 1), in which the intersection of drug targets and insomnia targets revealed 242 potential targets in total.

### 3.2 Construction of Network Pharmacology

**FIGURE 2:**
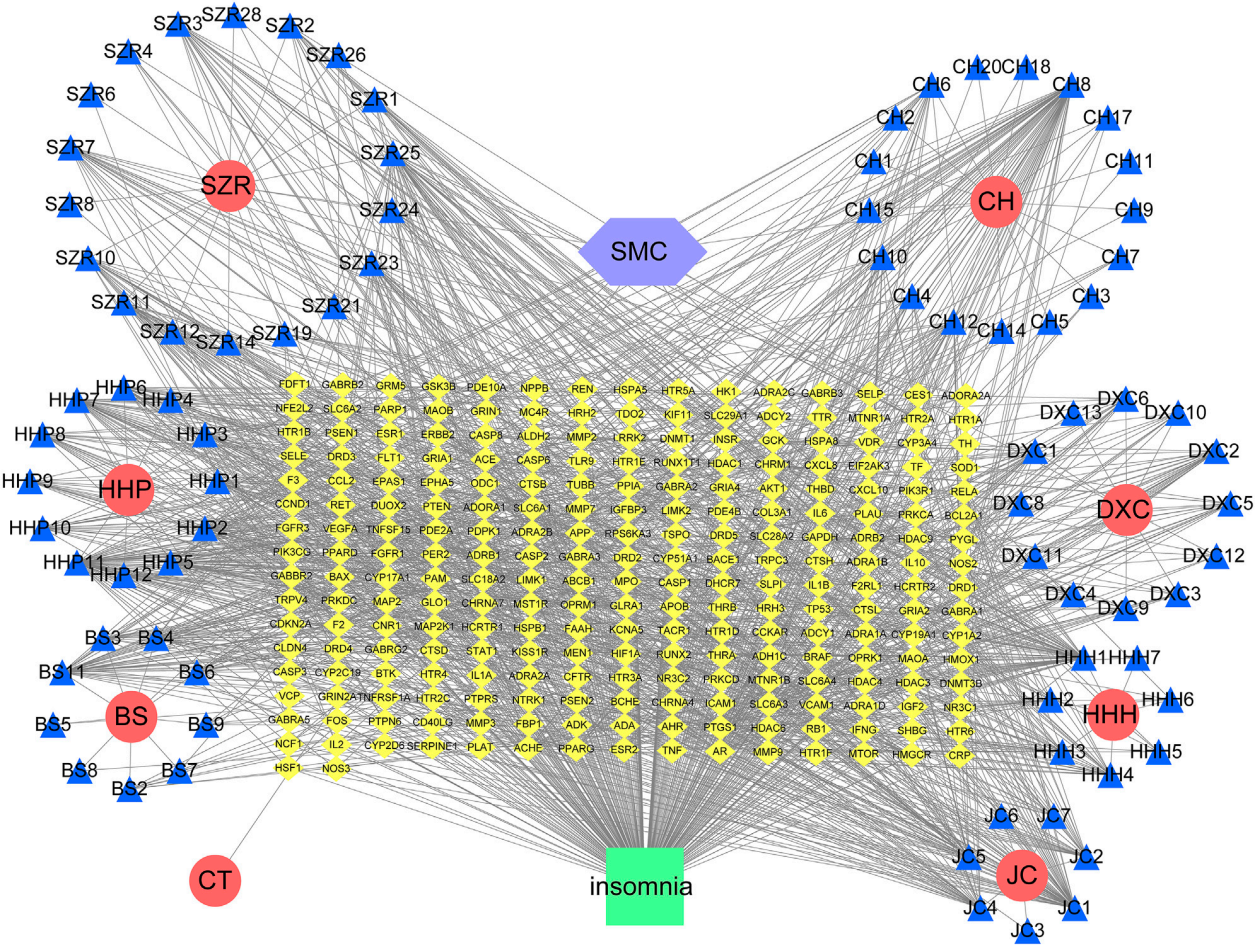
Network diagram of active ingredients of SMC and insomnia intersection targets. In the network, SZR represents Ziziphi Spinosae Semen, CH represents Bupleuri Radix, HHP represents Albiziae Cortex, DXC represents Junci Medulla, BS represents Paeoniae Radix Alba, HHH represents Albiziae Flos, JC represents *Bombyx batryticatus*, and CT represents Cicadae Periostracum.

The intersection network graph between disease and drug-active ingredients targets was constructed based on the relationship between each node and drug data (Figure 2). The graph showed 334 nodes and 1611 connections, while the relationship between nodes was represented by connections. The herbs were represented by their pinyin abbreviation, whose components’ abbreviation unified use of the abbreviation shown in Supplementary Table S1. The targets are marked with the abbreviation gene names in the Uniprot database.

**FIGURE 3:**
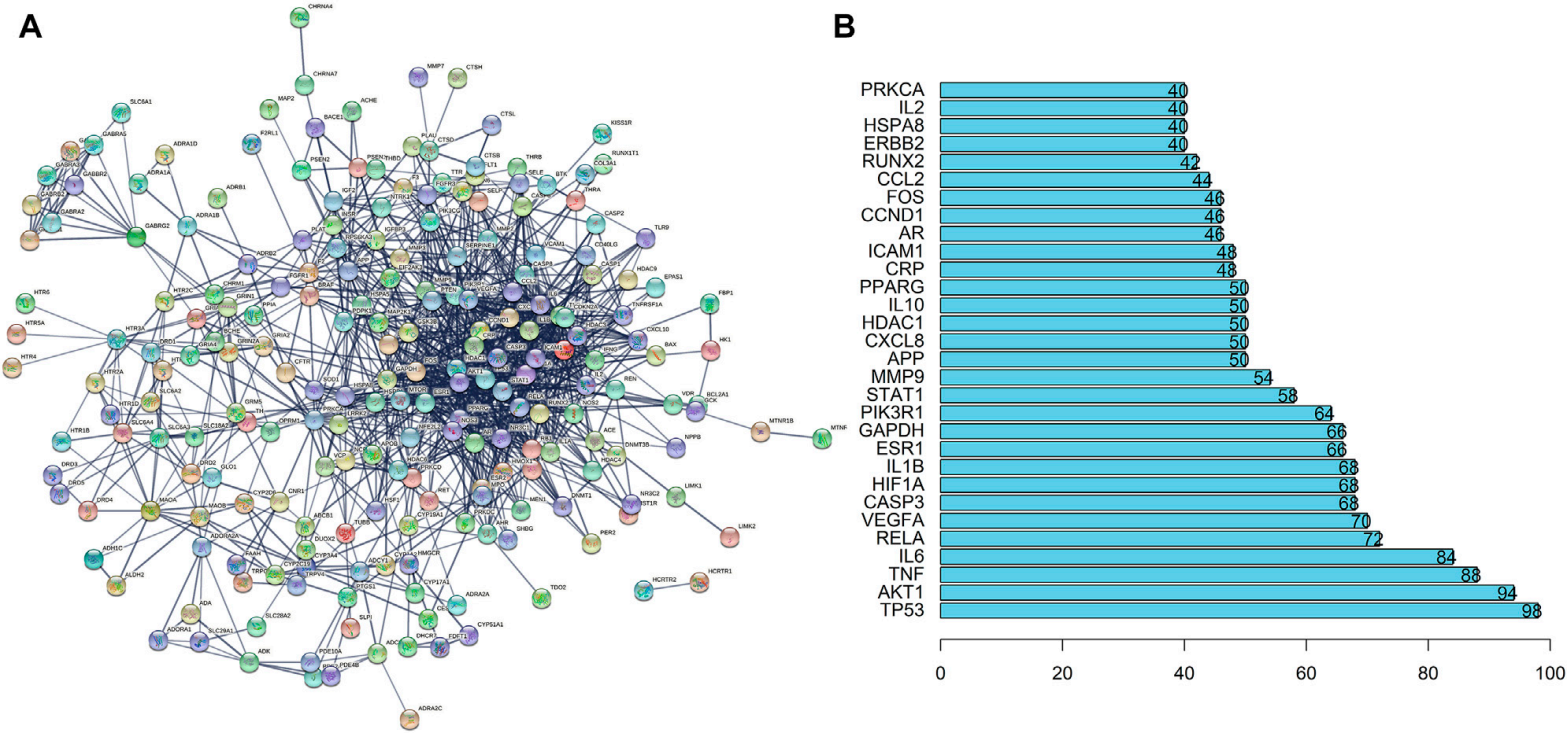
PPI Network Analysis of intersection targets. **(A)** PPI network of potential sleep disorder targets acted by major active components of SMC. There were 242 nodes and 1062 edges in the PPI network. **(B)** The top 30 genes in the PPI network.

Then, we imported the obtained 242 targets into the STRING website, setting “CONFIDENCE = 0.7”, the result revealed a total of 242 nodes and 1062 edges (Figure 3A). The histogram counted the detailed data in the result of the STRING website, in which each bar represented the number of edges corresponding to each node (Figure 3B).

### 3.3 GO and KEGG Pathway Enrichment Functional Analysis

**FIGURE 4:**
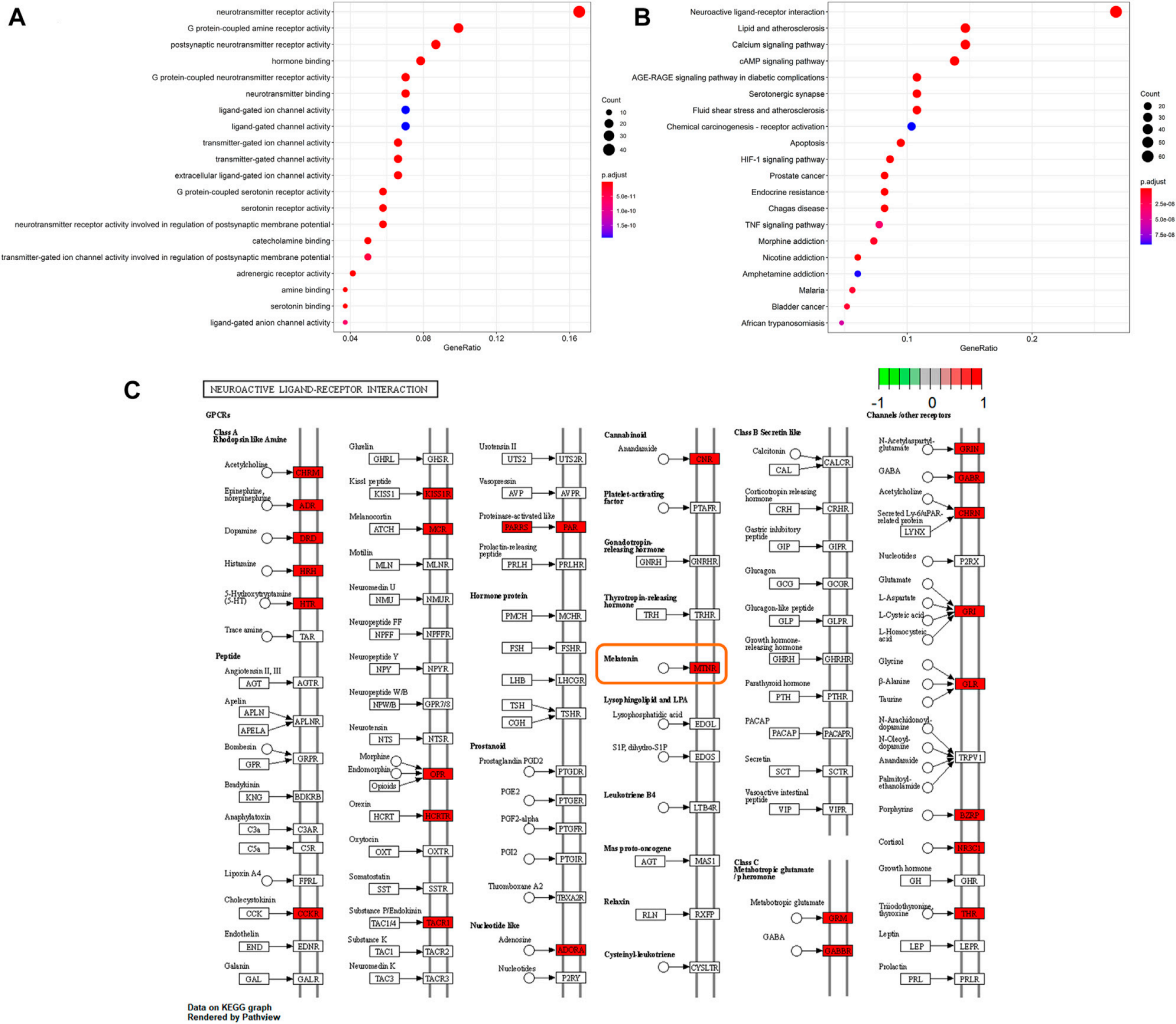
Statistics of functional and pathway enrichment. The x-axis represents the ratio number of genes, and the y-axis shows the pathway terms. The size of the circle represents gene count. Different color of circles represents different adjusted *p*-value. **(A)** Scatter plot of top 20 enriched GO functional analysis. **(B)** Scatter plot of top 20 enriched KEGG pathways. **(C)** Signal pathways in neuroactive ligand-receptor interaction based on the KEGG functional analysis.

GO enrichment functional analysis was performed as the bubble diagram using R software and the Bioconductor data package (Figure 4A). In the targets of GO enrichments, the top three biological processes were neurotransmitter receptor activity, G protein-coupled amine receptor activity, and postsynaptic neurotransmitter receptor activity, which indicated that neurotransmitters and receptors in the brain are the key factors in the regulatory process from drug to sleep disorder, reminding the potent neuroregulatory activities of SMC. The obtained genes were imported into KEGG pathway enrichment functional analysis using R software and the Bioconductor data package to obtain enriched pathways. The KEGG bubble diagram was performed by the enriched pathways (Figure 4B). Most of the enrichments were not related to sleep disorders according to other research reports, for example, lipid and atherosclerosis, fluid shear stress and atherosclerosis, as well as chemical carcinogenesis-receptor activation. In the top 20 enrichment pathways, neuroactive ligand–receptor interaction and serotonergic synapse indicated that intercellular crosstalk between neurons through ligand–receptor combination is an important process from drug to sleep disorder.

Neuroactive ligand–receptor interaction had shown a significant correlation between SMC and sleep disorder. Thus, we further searched for the signal pathways in this enrichment based on the KEGG functional analysis to explore the key targets between SMC and sleep disorder (Figure 4C). Many sleep-related receptors were included in these pathways, such as GABA receptors, 5-hydroxytryptophan receptors, histamine receptors, acetyl cholinergic receptors, orexin receptors, and melatonin receptors. Among these receptors, the melatonin receptor is closely related to biological rhythms and sleep homeostasis, becoming the focus of sleep research in recent years. Meanwhile, our previous KEGG pathway analysis results highlighted the enrichment of serotonergic synapse. Melatonin and orexin are important ligands highly associated with sleep and serotonergic synapse. Melatonin synthesis relies on serotonin as a precursor and is modulated by serotonin (Lee et al., 2021), while orexin was also reported to activate the OX1R and OX2R on serotonergic neurons (Xiao et al., 2021). Based on our network pharmacology analysis results and the published research reports, we designate the melatonin receptor and orexin receptor as the potential targets between SMC and sleep disorder.

### 3.4 SMC Improved Sleep and Depressive Symptoms in Sleep-Deprived Mice

**FIGURE 5:**
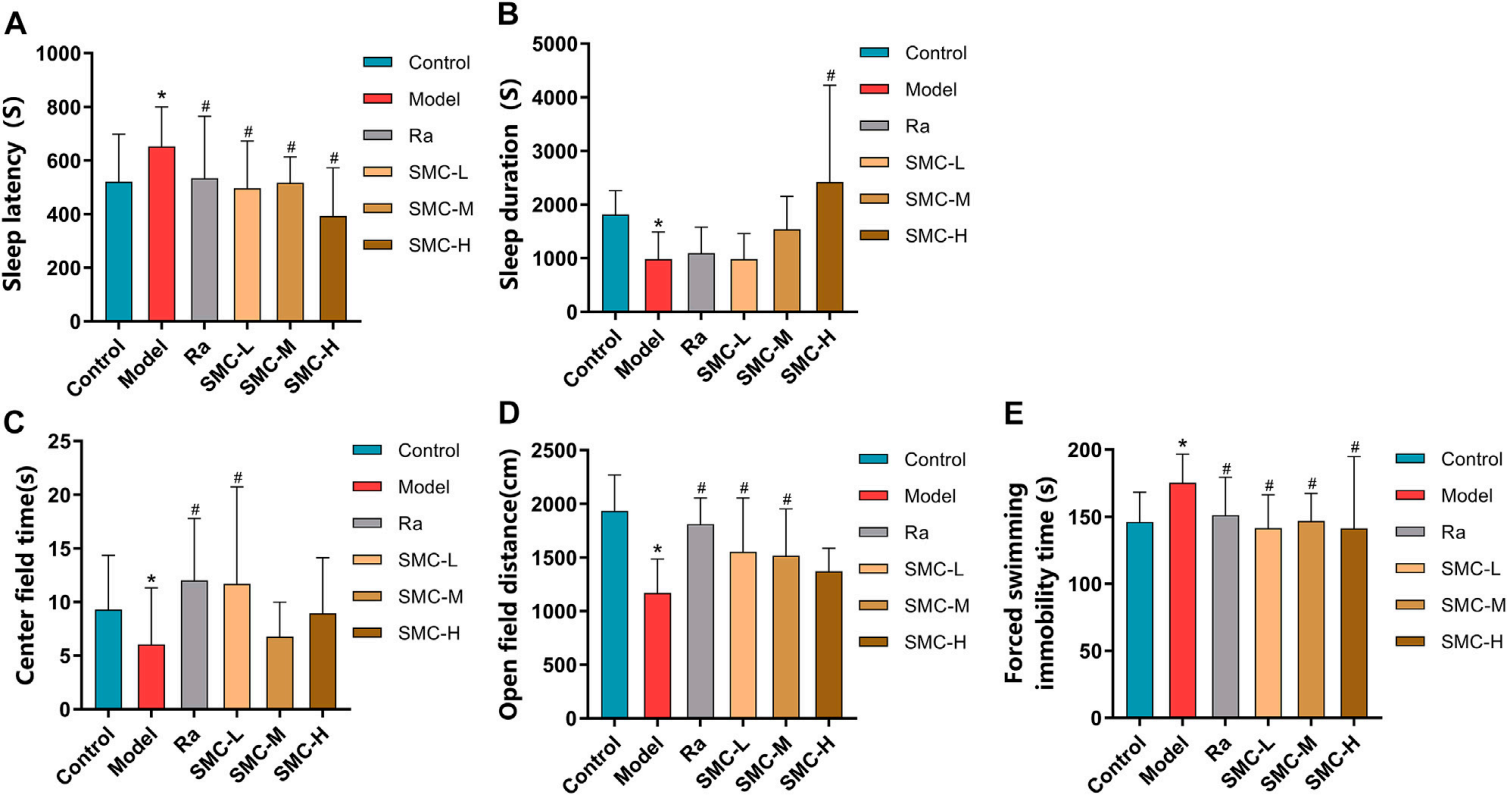
Behavioral experimental indexes of sleep-deprived mice after being treated with ramelteon and SMC (*n* = 11–14). **(A)** Sleep latency. **(B)** Sleep duration. **(C)** Center field time of OFT. **(D)** Open-field distance of OFT. **(E)** Immobility time of FST. *p < 0.05 *vs*. control group; #*p* < 0.05 *vs*. model group. All the data were represented as the mean ± SD.

To investigate the efficacies of SMC in treating the sleep disorder and depressive symptoms of sleep-deprived mice, mice were randomly arranged into control groups (control), sleep deprivation group (model), ramelteon (positive drug) treatment group (Ra), and three SMC treatment groups (SMC-L, SMC-M, and SMC-H) receiving 0.25 g/kg, 0.5 g/kg, and 1 g/kg of SMC, respectively. The pentobarbital-induced sleep experiment was performed to evaluate the hypnotic effects of SMC in sleep-deprived mice. As shown in Figure 5A, both SMC and Ra treatments significantly recovered the prolonged sleep latency induced by sleep deprivation (Figure 5A). No remarkable improvement in sleep duration was observed in Ra-treated mice; however, the high dose of SMC significantly improved the symptoms of sleep deprivation (Figure 5B).

Considering the interrelation between sleep deprivation and depression, we performed OFT to investigate if SMC improves mental behavior as previously described (Jin et al., 2019; Zhang N et al., 2020). As evident from Figures 5C,D, sleep-deprived mice showed less open-field distance and less stay in the center field than the control group (*p* < 0.05). Ra and the low dose of SMC significantly improved the two types of behavioral changes, and the middle dose of SMC enhanced open-field distance (*p* < 0.05). To verify the aforementioned findings, FST was also conducted to evaluate depressive-like behavior in rodents as previously described (Petit-Demouliere et al., 2005; Fan et al., 2021). Sleep-deprived mice exhibited longer forced swimming immobility time than the control group (*p* < 0.05), while treatment with ramelteon and three different doses of SMC exhibited significant improvement in the depressive-like behavior of mice (*p* < 0.05) (Figure 5E).

### 3.5 SMC Restores Melatonin Receptors and Orexin Receptor-Mediated Pathways in Sleep-Deprived Mice

**FIGURE 6:**
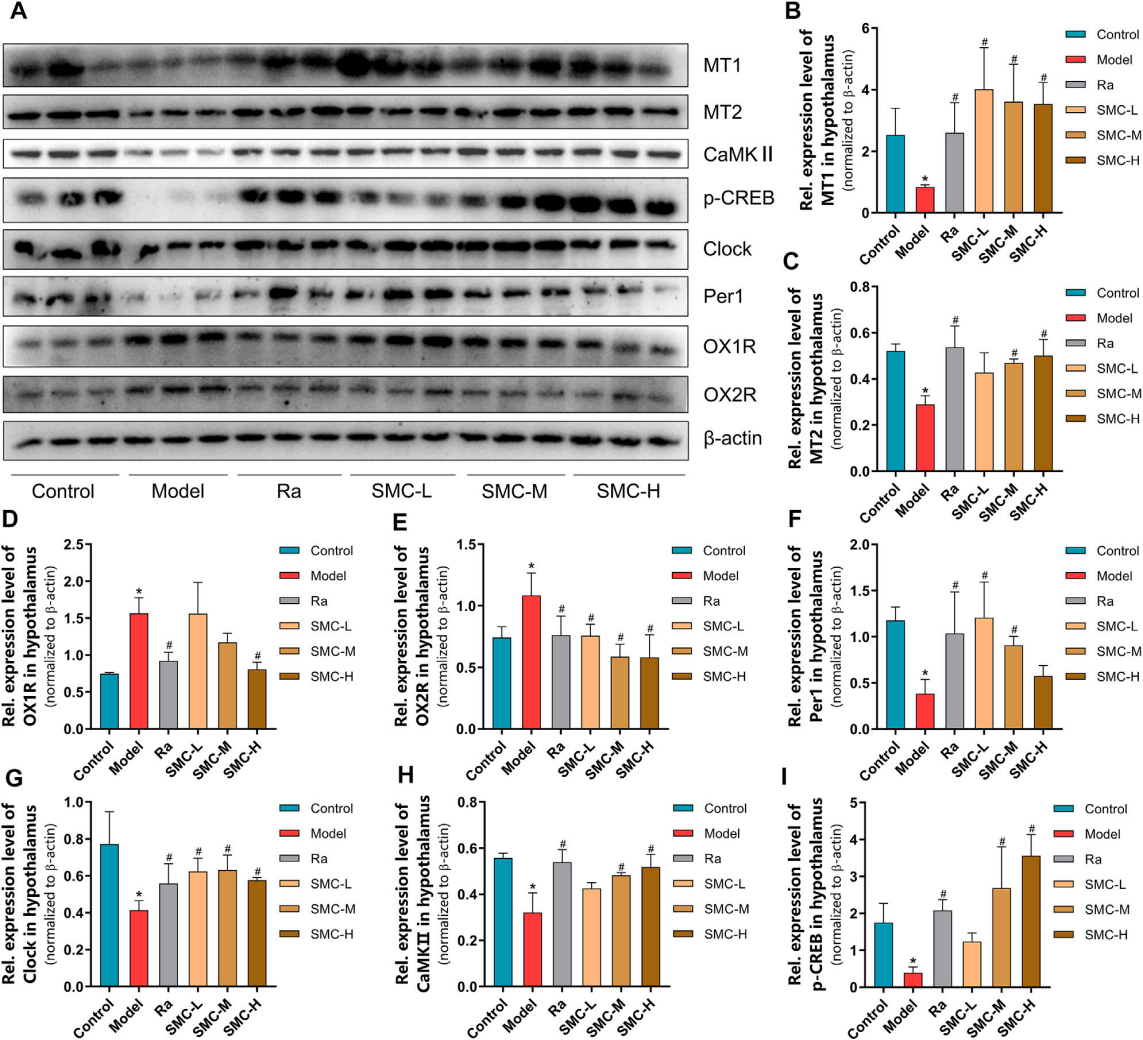
The changes of melatonin receptors and their related proteins in sleep-deprived mice after being treated with ramelteon and different doses of SMC (*n* = 3). **(A)** Western blotting results. Statistical data of the MT1 levels **(B)**, the MT2 levels **(C)**, the OX1R levels **(D)**, the OX2R levels **(E)**, the Per1 levels **(F)**, the clock levels **(G)**, the CaMKⅡ levels **(H),** and the p-CREB levels **(I)**. *p < 0.05 *vs*. control group; #*p* < 0.05 *vs*. model group. All the data were represented as the mean ± SD.

To verify the roles of melatonin receptors and orexin receptors in the hypnotic effect of SMC, we determined the expression levels of melatonin receptors (MT1 and MT2) and orexin receptors (OX1R and OX2R) in the hypothalamus of mice. Sleep deprivation decreased the expression levels of MT1 and MT2 and increased the expression levels of OX1R and OX2R (Figure 6A), while ramelteon and SMC treatments restored all the alterations (Figures 6B–E). We further determined the expression changes of MT-mediated downstream targets. clock genes Per1 (period circadian protein homolog 1) and clock (circadian locomotor output cycles protein kaput) are positively regarded as being regulated by MT1 (Jilg et al., 2005; Von Gall et al., 2005), while the activation of MT2 will lead to the upregulation of CaMKⅡ (Ca2+/calmodulin-dependent protein kinase II) and p-CREB (phosphor-cyclic AMP-responsive element-binding protein) (Wang et al., 2020). In our results, the levels of Per1, clock, CaMKⅡ, and p-CREB were significantly down-regulated by sleep deprivation and restored after the treatments with ramelteon or SMC (Figures 6A, F–I). These data demonstrated that SMC treatment abrogated the changes in MT1 and MT2 signaling pathways induced by sleep deprivation.

### 3.6 MT1 and MT2 Mediate the Hypnotic Effect of SMC Treatment

**FIGURE 7:**
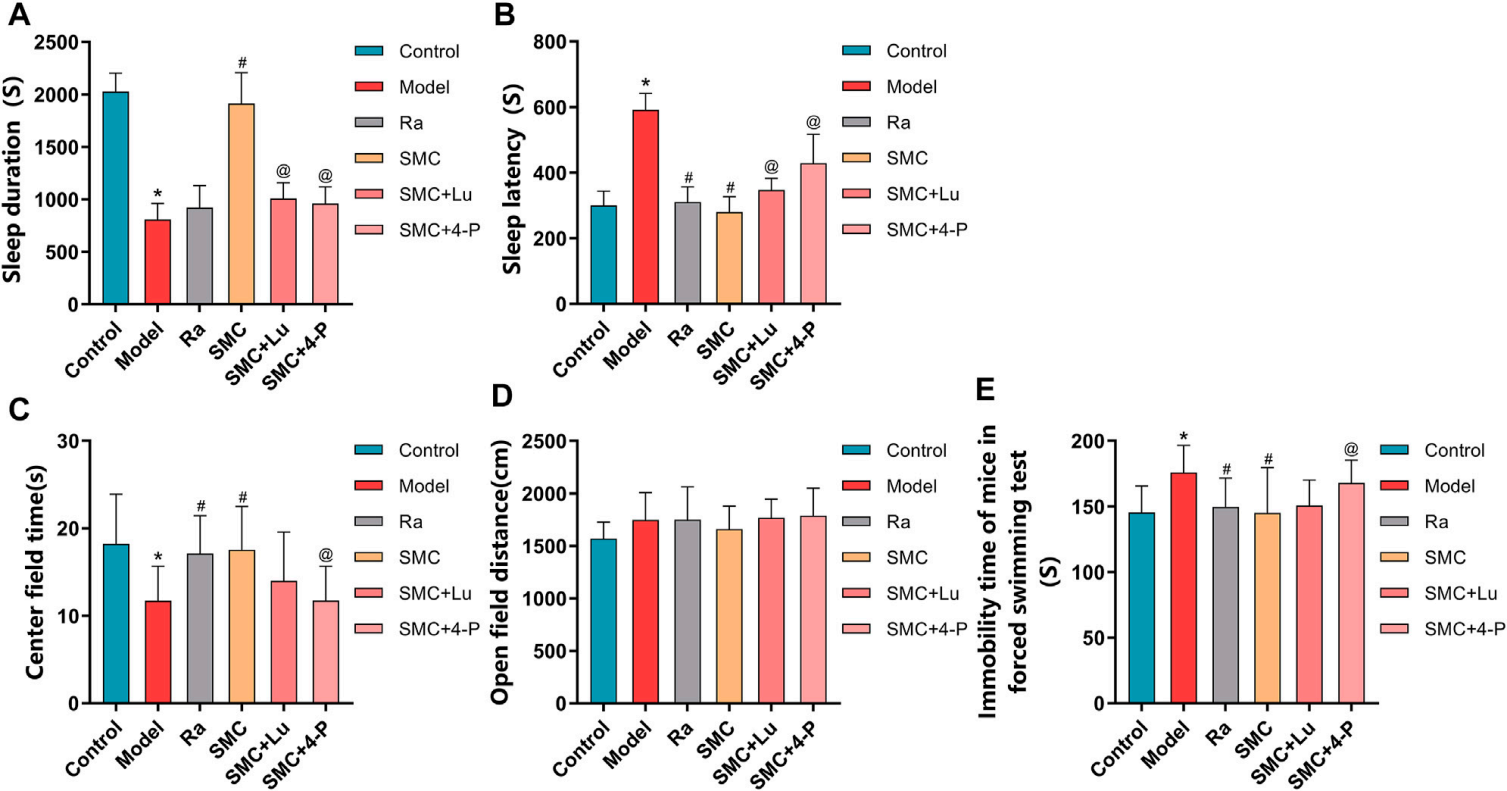
Behavioral experimental indexes of sleep-deprived mice after being treated with ramelteon, SMC, luzindole, and 4-P-PDOT (*n* = 12–14). **(A)** Sleep duration. **(B)** Sleep latency. **(C)** Center field time of OFT. **(D)** Open-field distance of OFT. **(E)** Immobility time of FST. *p < 0.05 vs. Control group; #*p* < 0.05 *vs*. Model group; @*p* < 0.05 *vs*. SMC group. All the data were represented as the mean ± SD.

To distinguish the roles of MT1 and MT2 in the hypnotic effect of SMC, sleep-deprived mice were randomly assigned to MT or MT2 antagonism prior to SMC treatment. In the pentobarbital-induced sleep experiment, non-selective MT antagonist luzindole and selective MT2 antagonist 4-P-PDOT significantly blocked the effects of SMC on sleep duration and sleep latency, while the antagonistic effect of 4-P-PDOT on sleep latency was more potent than luzindole (*p* < 0.05) (Figures 7A,B). For mental behaviors, MT2 antagonism by 4-P-PDOT attenuated the improvement of SMC on the time spent in the center field (*p* < 0.05), but no significant effect on open-field distance was observed for MT1 or MT2 antagonism (Figures 7C,D). FST also confirmed the involvement of MT2 in the therapeutic effect of SMC (Figure 7E).

**FIGURE 8:**
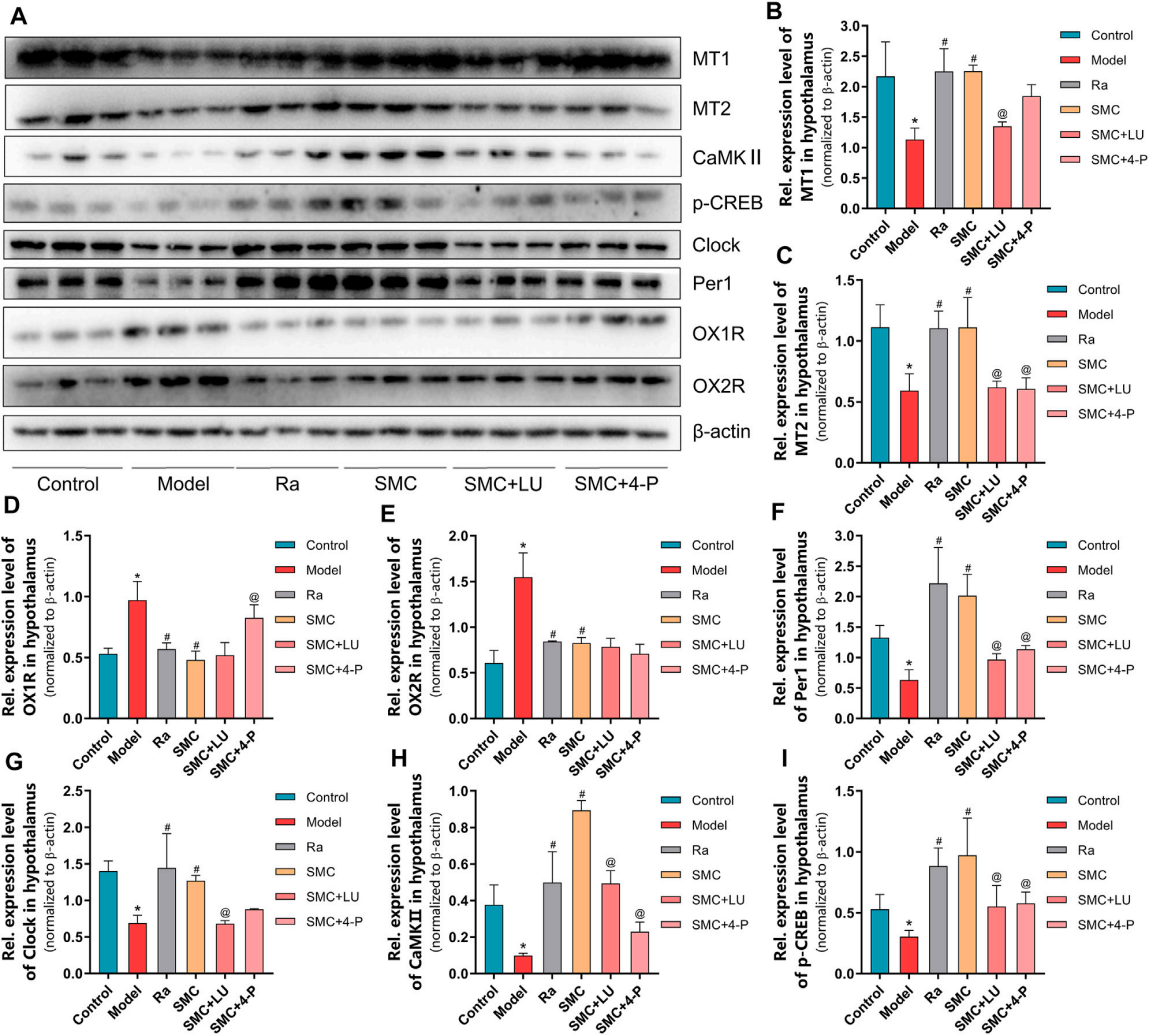
The changes of melatonin receptors and their related proteins in sleep-deprived mice after being treated with ramelteon, SMC, as well as melatonin receptor antagonists luzindole and 4-P-PDOT (*n* = 3). **(A)** Western blotting results. Statistical data of the MT1 levels **(B)**, the MT2 levels **(C)**, the OX1R levels **(D)**, the OX2R levels **(E)**, the Per1 levels **(F)**, the clock levels **(G)**, the CaMKⅡ levels **(H),** and the p-CREB levels **(I)**. *p < 0.05 *vs*. control group; #*p* < 0.05 *vs*. model group; @*p* < 0.05 *vs*. SMC group. All the data were represented as the mean ± SD.

To verify the aforementioned findings, we then examined the protein expression levels of melatonin receptors and orexin receptors. Compared with the SMC group, non-selective MT antagonist luzindole abrogated the enhancement of MT1 and MT2, while selective MT2 antagonist 4-P-PDOT only blocked the effect of SMC on MT2 (Figures 8A–C). Additionally, 4-P-PDOT suppressed the inhibition of SMC on OX1R but not OX2R (Figures 8D,E). For downstream signals of MT1 and MT2, luzindole significantly inhibited the changes of both MT1 and MT2 downstream targets, while 4-P-PDOT only significantly regulated Per1, CaMKⅡ, and p-CREB but not clock (Figures 8F–I), which might be attributed to the limited inhibitory effect of 4-P-PDOT to MT1.

## 4 Discussion

Long-term insufficient sleep brings about physiological, subjective experience, and consequent behavioral influences (Krause et al., 2017; Palmer and Alfano, 2017; Ben Simon et al., 2020). In regard to subjective experience, heightened emotional volatility and irritability, as well as reduced positive mood have been particularly associated with sleep deprivation (Ben Simon et al., 2020). Moreover, sleep disruption contributes to a higher probability of depression, anxiety, and suicide (Harvey et al., 2011; Bernert et al., 2015; Riemann et al., 2020). With regard to pathogenesis, insomnia and depression are also partly overlapped in multiple signal pathways, such as GABAergic and orexin (Michelson et al., 2014; Riemann and Spiegelhalder, 2014; Riemann et al., 2020).

SMC is commonly used for insomnia to improve anxiety, depression, and other symptoms. However, the mechanisms behind the therapeutic functions have not been fully elucidated. Network pharmacology is a promising method that combines multiple techniques and attempts to reveal potential mechanisms and relationships by constructing biological network models. At present, network pharmacology has been broadly applied to explore the molecular mechanism of TCM.

In the present study, a total of 242 target genes between SMC and insomnia were obtained after collecting and screening from multiple databases. To annotate the functions of these targets and related pathways, the GO enrichment and KEGG pathway enrichment analysis were further conducted. GO results showed that the top three biological processes were relevant to neurotransmitter receptor, G protein-coupled amine receptor, and postsynaptic neurotransmitter receptor pathways. We further searched the signal pathway to explore the critical targets of SMC for sleep disorder therapy and multiple receptors were included, such as GABA receptors, 5-HT receptors, histamine receptors, acetyl cholinergic receptors, orexin receptors, and MT receptors. Among these receptors, the MT receptors are closely related to biological rhythms and sleep homeostasis, making them the focus of sleep research in recent years. Based on our network pharmacology analysis results and the published research, we presumed that MT receptors as the potential targets of SMC.

Based on the findings of network pharmacology, *in vivo* experiments were performed to further validate the predictions. A mouse sleep-deprived model was adopted to generate sleep disorder and partly mental abnormality. Treatments with the MT receptor agonist ramelteon and SMC showed therapeutic efficacies. Activities of ramelteon have been well demonstrated, which is mainly related to the regulation of sleep latency (Sateia et al., 2017). SMC can improve both sleep latency and duration, indicating a different mechanism from that of ramelteon. In mental assessment, OFT has been generally used to evaluate anxiety and spontaneous activity levels (Jin et al., 2019; Zhang N et al., 2020), while FST is commonly used for depression and antidepressants screening (Petit-Demouliere et al., 2005; Fan et al., 2021). In the present study, significant differences appeared in FST rather than OFT, indicating the antidepressant effect of SMC and ramelteon. Although ramelteon has been widely used to treat sleep disorders with limited side effects, it is only applicable for sleep onset insomnia due to its single-target effect (Sateia et al., 2017). On the contrary, TCM is usually composed of multiple components, which work in a multi-target regulatory pattern. Herein, we demonstrated that SMC improved sleep latency and sleep duration, due to its regulatory activity including but not limited to melatonin receptors. Compared with the single effect of traditional melatonin receptor agonists on treating sleep onset insomnia, SMC exhibited therapeutic potential for various sleep disorders, such as sleep onset insomnia and sleep maintenance insomnia.

Both subtypes of melatonin receptors, MT1 and MT2, play important roles in mediating sleep homeostasis. MT1 is mainly regarded as a key receptor in regulating circadian rhythms. It has been reported that the oscillatory rhythm of Per1 and clock expression was severely damaged after the knockout of MT1 (Jilg et al., 2005; Von Gall et al., 2005). In mammals, transcriptional–translational loops of clock genes/proteins build up the circadian rhythm in the whole body (Harmer et al., 2001). Per1 and clock are two of the most important clock genes/proteins that participate in the transcriptional–translational loops and control the core circadian rhythm (Ripperger and Schibler, 2006; Asher and Schibler, 2011; Panda, 2016). Our results showed significant downregulation of MT1, Per1, and clock in sleep deprivation mice and recovery after SMC treatment. MT2 is thought to mainly regulate nonrapid eye movement sleep (NREM) (Gobbi and Comai, 2019). Evidence showed that MT2 activated by melatonin promotes the increase of intracellular calcium ions as well as the expression of CaMKII, reducing the phosphorylation level of CREB. As a result, the latency of NREM is reduced by restoring the inhibition of the N-methyl-D-aspartate receptor (NMDAR) which is the downstream factor of the MT2/Ca2+/CaMKⅡ/p-CREB pathway (Wang et al., 2020). Correspondingly, our study also revealed significant changes in the MT2/Ca2+/CaMKⅡ/p-CREB pathway, indicating that the reduced latency of NREM might be another influencing factor underlying SMC action.

Luzindole (N-0774) is a non-selective melatonin receptor antagonist, while 4-P-PDOT a potent, selective MT2 antagonist. In this research, the disparate selectivity of luzindole and 4-P-PDOT was utilized to discriminate the contributions of MT1 and MT2. The higher blocking efficacies for luzindole and 4-P-PDOT on Per1/clock and CaMKⅡ respectively were revealed due to the disparate selectivity in our results, which corresponds with the different signal transduction of MT1 and MT2 (Jilg et al., 2005; Von Gall et al., 2005; Wang et al., 2020). For behavioral experimental index, 4-P-PDOT showed higher blocking efficacies on sleep latency, center field time in OFT, and immobility time in FST in mice, while luzindole also revealed blocking efficacies to some extent. This phenomenon indicated that the therapeutic efficacy of SMC on sleep and mental disorder is via the combined effect of MT1 and MT2, while the MT2 showed a stronger mediational effect than MT1.

Recent research indicated that there was a regulatory relationship between MT and orexin, where MT inhibited orexin neurons in the perifornical lateral hypothalamus through the MT1 receptors and promoted sleep (Sharma et al., 2018). Our results showed that the expression levels of OX1R and OX2R increased in sleep deprivation mice, while ramelteon and SMC treatments restored the alterations. Additionally, the selective MT2 antagonist 4-P-PDOT suppressed the inhibition of SMC on OX1R but not OX2R. These results indicated that the regulation of OX1R by SMC treatment is mainly through MT receptors, while the regulation of OX2R by SMC in other different pathways.

Frankly, the present study has several limitations. First, the targets of insomnia and SMC from online databases were based on the predicted data. Thus, those unproven and undocumented targets may be omitted. Second, whether SMC treatment regulates MT receptors and their related signaling pathways directly still remains unclear. Third, the alterations of MT receptors and other related proteins were found in the hypothalamus, while other brain regions have not been determined. Consequently, additional research is required to further explore the molecular mechanisms of SMC in treating insomnia.

Taking these together, SMC improves sleep disorder and mental symptoms by regulating MT1 and MT2 and their related pathways in sleep-deprived mice and our results provide the theoretical support for the clinical application and potential expansion.

## Data Availability

The original contributions presented in the study are included in the article/Supplementary Material; further inquiries can be directed to the corresponding authors.
