# Supplementary material for: Shumian Capsule Improves the Sleep Disorder and Mental Symptoms Through Melatonin Receptors in Sleep-Deprived Mice
Source: Front Pharmacol. 2022 Jul 8;13:925828. doi: 10.3389/fphar.2022.925828 (PMC9304889; doi:10.3389/fphar.2022.925828)
Supplement: Supplementary file 1 [file DataSheet1.pdf]

Supplementary Material

Supplementary Table 1. Key active ingredients in Shumian capsule.

**Herbs**	**Chemicals**	**Resource**	**abbreviation**
Ziziphi Spinosae Semen	(S)-Coclaurine	TCMSP	SZR1
Ziziphi Spinosae Semen	n-methylasimilobine	TCMSP	SZR2
Ziziphi Spinosae Semen	dl-Nuciferine	TCMSP	SZR3
Ziziphi Spinosae Semen	zizyphusine	TCMSP	SZR4
Ziziphi Spinosae Semen	jujuboside A_qt	TCMSP	SZR5
Ziziphi Spinosae Semen	eleutheroside A	TCMSP	SZR6
Ziziphi Spinosae Semen	jujuboside B_qt	TCMSP	SZR7
Ziziphi Spinosae Semen	swertisin	TCMSP	SZR8
Ziziphi Spinosae Semen	Daucosterol	TCMSP	SZR9
Ziziphi Spinosae Semen	phytosterol	TCMSP	SZR10
Ziziphi Spinosae Semen	alphitolic acid	TCMSP	SZR11
Ziziphi Spinosae Semen	ceanothic acid	TCMSP	SZR12
Ziziphi Spinosae Semen	sanjoinine D	TCMSP	SZR13
Ziziphi Spinosae Semen	Mairin	TCMSP	SZR14
Ziziphi Spinosae Semen	sanjoinenine	TCMSP	SZR15
Ziziphi Spinosae Semen	sanjoinine A	TCMSP	SZR16

Ziziphi Spinosae Semen	sanjoinine B	TCMSP	SZR17
Ziziphi Spinosae Semen	Spinosin	TCMSP	SZR18
Ziziphi Spinosae Semen	Jujubogenin	YaTCM	SZR19
Ziziphi Spinosae Semen	Ursolic acid	YaTCM	SZR20
Ziziphi Spinosae Semen	daucosterin	YaTCM	SZR21
Ziziphi Spinosae Semen	Ferulic acid	YaTCM	SZR22
Ziziphi Spinosae Semen	Cearoin	YaTCM	SZR23
Ziziphi Spinosae Semen	N-cis-Feruloyltyramine	YaTCM	SZR24
Ziziphi Spinosae Semen	(+)-1(R)-Coclaurine	YaTCM	SZR25
Ziziphi Spinosae Semen	(-)-Caaverine	YaTCM	SZR26
Ziziphi Spinosae Semen	Juzirine	YaTCM	SZR27
Ziziphi Spinosae Semen	Coumestrol	YaTCM	SZR28
Ziziphi Spinosae Semen	Dauricine	YaTCM	SZR29
Bupleuri Radix	Linoleyl acetate	TCMSP	CH1
Bupleuri Radix	Sainfuran	TCMSP	CH2
Bupleuri Radix	Cubebin	TCMSP	CH3
Bupleuri Radix	isorhamnetin	TCMSP	CH4
Bupleuri Radix	Areapillin	TCMSP	CH5
Bupleuri Radix	Octalupine	TCMSP	CH6
Bupleuri Radix	Longikaurin A	TCMSP	CH7

Bupleuri Radix	quercetin	TCMSP	CH8
Bupleuri Radix	(+)-Anomalin	TCMSP	CH9
Bupleuri Radix	Stigmasterol	TCMSP	CH10
Bupleuri Radix	α-spinasterol	TCMSP	CH11
Bupleuri Radix	kaempferol	TCMSP	CH12
Bupleuri Radix	Baicalin	TCMSP	CH13
Bupleuri Radix	3,5,6,7-tetramethoy-2-(3,4,5-trimethoxyphenyl) chromone	TCMSP	CH14
Bupleuri Radix	Troxerutin	TCMSP	CH15
Bupleuri Radix	saikosaponin c_qt	TCMSP	CH16
Bupleuri Radix	petunidin	TCMSP	CH17
Bupleuri Radix	Spinasterol	ETCM	CH18
Bupleuri Radix	Scopolin	ETCM	CH19
Bupleuri Radix	Sabinene Hydrate	ETCM	CH20
Albiziae Flos	quercetin	Research articles	HHH1
Albiziae Flos	isorhamnetin	Research articles	HHH2
Albiziae Flos	luteolin	Research articles	HHH3
Albiziae Flos	kaempferol	Research articles	HHH4

Albiziae Flos	Chryseriol	Research articles	HHH5
Albiziae Flos	taxifolin	Research articles	HHH6
Albiziae Flos	α-spinasterol	Research articles	HHH7
Albiziae Flos	ergosterol peroxide	Research articles	HHH8
Albiziae Cortex	Acacic Acid Lactone	ETCM	HHP1
Albiziae Cortex	Julibrotriterpenoidal Lactone A	ETCM	HHP2
Albiziae Cortex	Machaerinic Acid Lactone	ETCM	HHP3
Albiziae Cortex	Machaerinic Acid Methyl Ester	ETCM	HHP4
Albiziae Cortex	Noradrenaline	ETCM	HHP5
Albiziae Cortex	trihydroxyflavone	ETCM	HHP6
Albiziae Cortex	julibrotriterpenoidal lactone A	ETCM	HHP7
Albiziae Cortex	5 ,5′ dimethoxy 7 oxolariciressinol	Research articles	HHP8
Albiziae Cortex	Syringaresino	Research articles	HHP9
Albiziae Cortex	(R) - 2-trans-2,6-dimethyl -6-o - β - d-glycopyranosyl -2,8-octadienoic acid	Research articles	HHP10

Albiziae Cortex	quercetin	Research articles	HHP11
Albiziae Cortex	beta-sitosterol	Research articles	HHP12
Albiziae Cortex	Daucosterol	Research articles	HHP13
Paeoniae Radix Alba	benzoyl paeoniflorin	TCMSP	BS1
Paeoniae Radix Alba	sitosterol	TCMSP	BS2
Paeoniae Radix Alba	beta-sitosterol	TCMSP	BS3
Paeoniae Radix Alba	kaempferol	TCMSP	BS4
Paeoniae Radix Alba	(3S,5R,8R,9R,10S,14S)-3,17-dihydroxy-4,4,8,10,14-pentamethyl-2,3,5,6,7,9-hexahydro-1H-cyclopenta[a]phenanthrene-15,16-dione	TCMSP	BS5
Paeoniae Radix Alba	Lactiflorin	TCMSP	BS6
Paeoniae Radix Alba	paeoniflorin	TCMSP	BS7
Paeoniae Radix Alba	(+)-catechin	TCMSP	BS8
Paeoniae Radix Alba	Mairin	TCMSP	BS9
Paeoniae Radix Alba	11alpha,12alpha-epoxy-3beta-23-dihydroxy-30-norolean-20-en-28,12beta-olide	TCMSP	BS10

Paeoniae Radix Alba	albiflorin_qt	TCMSP	BS11
Paeoniae Radix Alba	paeoniflorin_qt	TCMSP	BS12
Paeoniae Radix Alba	paeoniflorgenone	TCMSP	BS13
Cicadae Periostracum	acetamide	TCMSP	CT
Bombyx Batryticatus	quercetin	Research articles	JC1
Bombyx Batryticatus	kaempferol	Research articles	JC2
Bombyx Batryticatus	sitosterol	Research articles	JC3
Bombyx Batryticatus	3,5-Dihydroxyergosta-7,22-dien-6-one	Research articles	JC4
Bombyx Batryticatus	3-benzyl-6-isopropyl-4-methyl-2,5-morpholine Dione	Research articles	JC5
Bombyx Batryticatus	3-hydroxy kynurenine	Research articles	JC6
Bombyx Batryticatus	Ergosterol endoperoxide	Research articles	JC7
Junci Medulla	luteolin	TCMSP	DXC1
Junci Medulla	2,8-dihydroxy-1,7-dimethyl-6-ethenyl-10,11-dihydrodibenz[b,f]-oxepin	TCMSP	DXC2
Junci Medulla	5-(1-methoxyethyl)-2,6-dihydroxy-1,7-dimethyl-	TCMSP	DXC3

	9,10-dihydrophenanthrene		
Junci Medulla	8-hydroxy-2-methoxy- 1,6-dimethyl-5-ethenyl- 9,10-dihydrophenanthrene	TCMSP	DXC4
Junci Medulla	2-hydroxy-7-methoxy-1,8- dimethyl-5-ethenyl-9,10- dihydrophenanthrene	TCMSP	DXC5
Junci Medulla	1,8-dimethyl-4-vinyl-9,10- dihydrophenanthrene-2,7- diol	TCMSP	DXC6
Junci Medulla	2,8-dihydroxy-1,6-dimethyl- 5- ethenyl-9,10- dihydrophenanthrene	TCMSP	DXC7
Junci Medulla	hederagenin	TCMSP	DXC8
Junci Medulla	7-hydroxy-8-methyl-4- vinyl-9,10- dihydrophenanthrene-1- carboxylic acid	TCMSP	DXC9
Junci Medulla	1-hydroxyethyl)-2,6- dihydroxy-1,7- dimethyl-9,10- dihydrophenanthrene	TCMSP	DXC10
Junci Medulla	dehydroeffusal	TCMSP	DXC11
Junci Medulla	3,7-dihydroxy-2,8-dimethyl- 9,10-	TCMSP	DXC12

	dihydrophenanthrene-4-carbaldehyde		
Junci Medulla	sitosterol	TCMSP	DXC13
